# When X Does Not Mark the Spot: Autosomal Dominant and Recessive Forms of Renal Hypophosphatemic Rickets and Osteomalacia

**DOI:** 10.1007/s11914-026-00963-4

**Published:** 2026-07-07

**Authors:** Carlos R. Ferreira, Erik A. Imel

**Affiliations:** 1 Unit On Skeletal Genomics, Eunice Kennedy Shriver National Institute of Child Health and Human Development, National Institutes of Health, 10 Center Drive, Building 10 CRC, Room 2-5142, Bethesda, MD 20892 USA; 2 Departments of Medicine and Pediatrics, Endocrinology, Indiana University School of Medicine, 1120 West Michigan Street, CL 380, Indianapolis, IN 46112 USA

**Keywords:** Autosomal dominant hypophosphatemic rickets, Autosomal recessive hypophosphatemic rickets, Fibroblast growth factor 23, Iron, Osteomalacia

## Abstract

**Purpose of Review:**

Conditions resulting in elevated fibroblast growth factor 23 (FGF23) cause hypophosphatemic rickets and osteomalacia. The most common of these is X-linked hypophosphatemia. In this review we will broadly discuss the other less common and clinically distinct forms of renal hypophosphatemia, with a focus on the autosomal dominant and autosomal recessive types.

**Recent Findings:**

Variants in multiple genes cause dominant (*FGF23, SGK3, FGFR1*), recessive (*DMP1, ENPP1, FAM20C*, *INPPL1*) or even somatic (*NRAS, HRAS, GNAS*, gene fusions) conditions of FGF23 excess, with important phenotypic differences. For example, in autosomal dominant hypophosphatemic rickets due to *FGF23* variants, iron deficiency drives the phenotype, while *ENPP1* variants cause phenotypes ranging from severe neonatal vascular calcifications to rickets or osteoporosis. Other gene abnormalities cause FGF23-independent hypophosphatemia, often involving kidney disease.

**Summary:**

Recognizing the different mechanisms and phenotypes of hypophosphatemic conditions is critical to prognosis, management and to developing more effective therapies.

## Introduction

The most commonly encountered forms of rickets and osteomalacia are nutritional, primarily due to deficiencies in intake or absorption of vitamin D or calcium [1]. However, the hypophosphatemic forms of rickets and osteomalacia have importance as they have influenced our understanding of phosphate metabolism and differ in treatment strategies. Furthermore, understanding the differences among these forms of rickets are important for accurate diagnosis, management, and counseling of patients and families.

Of the hypophosphatemic forms of rickets and osteomalacia, X-linked hypophosphatemia (XLH) is the most common [2]. However, several other genetic forms of hypophosphatemia should be considered as the disease consequences and treatments vary across different genetic causes. The goal of this review is to broadly discuss the other genetic (as well as non-genetic) forms of hypophosphatemic rickets and osteomalacia, with a focus on the autosomal dominant and autosomal recessive forms.

## Phosphorus Regulation and FGF23

Phosphorus in the body exists in the form of organic phosphate (such as phospholipids, nucleic acids, or ATP) or inorganic phosphate (such as free phosphate ions or phosphate salts, e.g., hydroxyapatite) [2]. Phosphate is a major component of the skeleton, as well as being critical to several biologic processes including cell signaling pathways, energy metabolism, DNA and RNA. The normal blood levels of phosphate vary across ages with levels in infancy and early childhood being much higher than in adults. Hypophosphatemia occurs due to three main mechanisms: acute transfer of phosphate from the extracellular to the intracellular space, acute or chronic impairments of phosphate intake or its gastrointestinal absorption, or renal losses of phosphate [2]. When hypophosphatemia is chronic, it results in osteomalacia and rickets, along with other musculoskeletal consequences.

Renal forms of hypophosphatemia represent the most common of the genetic hypophosphatemias and may occur due either to hormone signaling pathways or due to renal tubular damage, both impairing the reabsorption of phosphate in the proximal renal tubule. The primary hormones involved in regulating phosphate reabsorption are parathyroid hormone (PTH) and fibroblast growth factor 23 (FGF23) [2]. PTH is produced in the parathyroid glands, primarily regulated by the response of the calcium sensing receptor (CaSR) to serum calcium, and also to effects of 1,25-dihydroxyvitamin D (1,25(OH)_2_D; the active form of vitamin D). FGF23 is produced mainly in osteocytes in response to 1,25(OH)_2_D and serum phosphate concentrations. FGF23 interacts with the complex of FGF receptors and Klotho in the renal tubule. The effects of both PTH and FGF23 downregulate phosphate reabsorption through the sodium phosphate co-transporters (NPT2a and NPT2c) and thus excess levels of these hormones promote the development of renal hypophosphatemia [2]. FGF23 also impairs the production of 1,25(OH)_2_D (through 1-⍺-hydroxylase), and upregulates 1,25(OH)_2_D catabolism (through 24-hydroxylase).

Although frequently referred to as “hypophosphatemic rickets” based on the childhood presentations, these hypophosphatemic disorders manifest at any age with effects on pediatric growth and skeletal development (rickets and osteomalacia) or in adulthood with osteomalacia, bone pain, insufficiency fractures, and other musculoskeletal problems. Furthermore, several of these conditions also manifest with dental and joint-related abnormalities. XLH is X-linked dominant and occurs due to pathogenic variants in *PHEX* (phosphate regulating endopeptidase, X-linked) leading to increased gene expression and circulating levels of FGF23. However, various autosomal forms of hypophosphatemic rickets occur due to changes in FGF23 expression, production, degradation or responsiveness, or via direct renal tubular damage Table 1.

**Table 1:** Renal hypophosphatemias

FGF23 mediated	FGF23 independent
Autosomal dominant	Autosomal dominant
ADHR –* FGF23*	Peroxisomal L-bifunctional protein deficiency – *EHADDH*
ADHR – *SGK3*	Arginine:glycine amidinotransferase deficiency deficiency – *GATM*
Osteoglophonic dysplasia – *FGFR1*	
Autosomal recessive	Autosomal recessive
ARHR1 – *DMP1*	Nephropathic cystinosis – *CTNS*
ARHR2 –* ENPP1*	Leigh syndrome/Acadian variant Fanconi syndrome – *NDUFAF6*
ARHR3 – *FAM20C*	Hereditary hypophosphatemic rickets with hypercalciuria – SLC34A3*
Opsismodysplasia – *INPPL1*	Infantile hypercalcemia with phosphaturia – SLC34A1*
X-linked	X-linked
XLH– *PHEX*	Lowe syndrome - *OCRL*
	Dents disease - *CLCN5*
	Mitochondrial disorders
Somatic	
FD/MAS – *GNAS*	
Cutaneous-skeletal hypophosphatemia syndrome – *NRAS, HRAS*	
Tumor-induced osteomalacia – *FN1:FGFR1* fusion – *FN1:FGF1 fusion* – Gene *rearrangements involving Klotho* – V*arious malignancies*	
Non-genetic	Non-genetic
Iron – intravenous infusions	Drug induced
Autoimmune antibodies to PHEX	
Cadmium toxicity	
Acute hepatitis	
Biliary atresia	
Post hepatic resection	
Post renal transplant	

^*^Heterozygotes with variants in *SLC34A1 or SLC34A3 may manifest phosphaturia and hypercalciuria as well*

## Autosomal Dominant Hypophosphatemic Rickets (ADHR) – FGF23 Mediated

### *FGF23 *Variant ADHR

*FGF23* was originally discovered as the causative gene during analysis of kindreds having an autosomal dominant hypophosphatemic rickets pattern. These initial kindreds had pathogenic variants resulting in replacing arginine with other amino acids (glutamine or tryptophan) at residues 176 or 179, occuring in a proteolytic cleavage motif (RXXR^179^/S^180^) in the FGF23 protein [3–5]. More recently the number of variants impairing FGF23 cleavage was expanded when two different groups reported patients from families having ADHR due to pathogenic variants replacing the serine at position 180: one causing a change from serine 180 to isoleucine, and one replacing serine 180 with arginine [6, 7]. Each of these amino acid alterations in this location result in impaired cleavage of FGF23 between amino acid 179 and 180 [4]. Under normal circumstances, a portion of intact FGF23 is cleaved into inactive N-terminal and C-terminal fragments before secretion from the cell. In ADHR, the *FGF23* variants make a protein that is resistant to cleavage, which results in elevations of intact FGF23 and subsequent hypophosphatemia. However, there is evidence that the body is sometimes able to appropriately downregulate FGF23 to maintain normal phosphate levels. In ADHR, there is incomplete penetrance, and variable timing of expressivity [3, 8]. Some patients present in childhood with rickets and lower limb deformities, only to later spontaneously normalize phosphate homeostasis. Other patients harbouring the same variants are normophosphatemic through childhood and at a later time in adolescence or adulthood newly begin to manifest hypophosphatemia. The phosphate status correlates with the serum intact FGF23 concentration at the time of measurement. Those with delayed onset of hypophosphatemia have developed elevated intact FGF23 concentrations, while those that normalize their FGF23 levels also normalize their phosphate concentrations [3, 8].

The waxing and waning of FGF23 levels (and hence phosphate) in ADHR is due to alterations in iron stores. Low iron concentrations cause elevated *FGF23* gene expression; but typically the FGF23 protein produced is cleaved so that intact FGF23 remains normal [9]. However, in ADHR the FGF23 amino acid change at the cleavage motif impairs its cleavage so that intact FGF23 concentrations increase when iron concentration or stores are low, driving higher *FGF23* gene expression [9, 10]. This then results in FGF23-mediated hypophosphatemia.

**Fig. 1:**
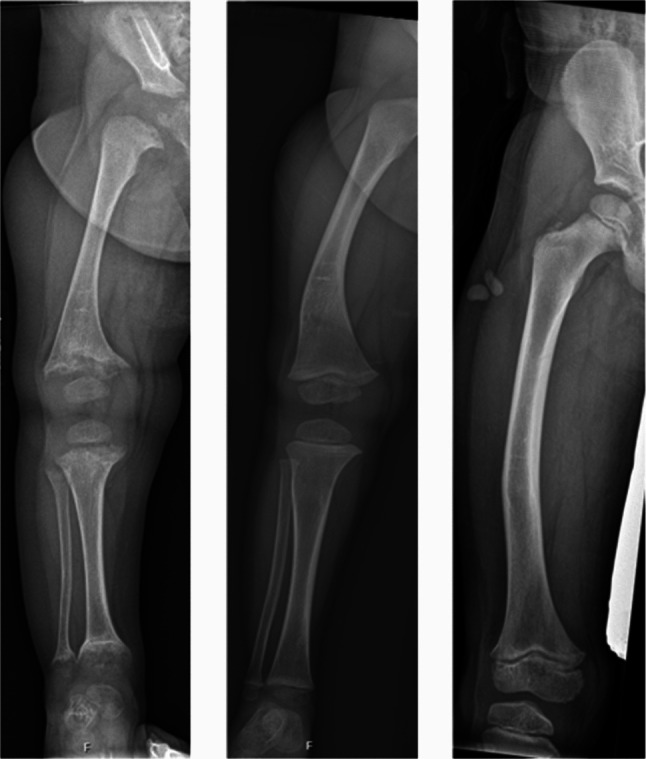
A male child with ADHR, due to pathogenic variant in FGF23, demonstrated rickets and hypophosphatemia at 22 months of age (left), with improvement at 32 months of age (middle) after about 6 months of calcitriol and phosphate treatment, and with resolution of rickets and hypophosphatemia at 5 years 10 months (right) after iron deficiency was corrected with oral iron. Figure reproduced from Davis K, Imel EA, and Kelley J., Hypophosphatemic rickets and short stature, J Bone Miner Res. 2024;39(7):821–825, by permission of Oxford University Press and the American Society for Bone and Mineral Research[13]

Repleting iron stores with oral forms of iron normalized the intact FGF23 and phosphate in a small ADHR clinical trial, as well as in other case reports, including patients having variants in each of the reported amino acid locations R176, R179, or S180 [6, 7, 11–13]. Figure 1 demonstrates this clinical resolution of rickets during iron repletion. Thus, oral iron repletion is a better treatment option for the iron-deficient patient with ADHR, though some may still require calcitriol and phosphate transiently for healing of their rickets or osteomalacia. However, in ADHR we would not recommend burosumab (a monoclonal antibody to FGF23 approved for XLH and tumor-induced osteomalacia [TIO]) due to its high cost and the potential to normalize phosphate metabolism without such treatment once iron stores are repleted. However, in ADHR we also do not recommend intravenous iron as the source for repletion. This is because multiple forms of intravenous iron cause an acute elevation of intact FGF23 levels, to a degree that causes hypophosphataemia in patients without ADHR (see section below).

### *SGK3* Variant ADHR

Hypophosphatemia in an autosomal dominant inheritance pattern has also been ascribed to *SGK3* pathogenic variants in one kindred with five affected family members [14]. SGK3 is a renal protein that impairs sodium phosphate transporter activity, so one would expect this variant to cause FGF23-independent hypophosphatemia. Indeed, in mouse models of SGK3 deficiency, FGF23 levels are low [15]. However, FGF23 levels did appear to be dysregulated in affected subjects tested in this kindred, with intact FGF23 in a range of 42–145 pg/ml despite hypophosphatemia [14]. These values during hypophosphatemia are higher than the 27 or 30 pg/ml threshholds other authors have suggested as supporting FGF23-mediated hyposphatemia [16, 17]. The mechanism or degree of FGF23 dysregulation, if present, remains uncertain in the setting of *SGK3* variants.

### *FGFR1* Variant Osteoglophonic Dysplasia

**Fig. 2:**
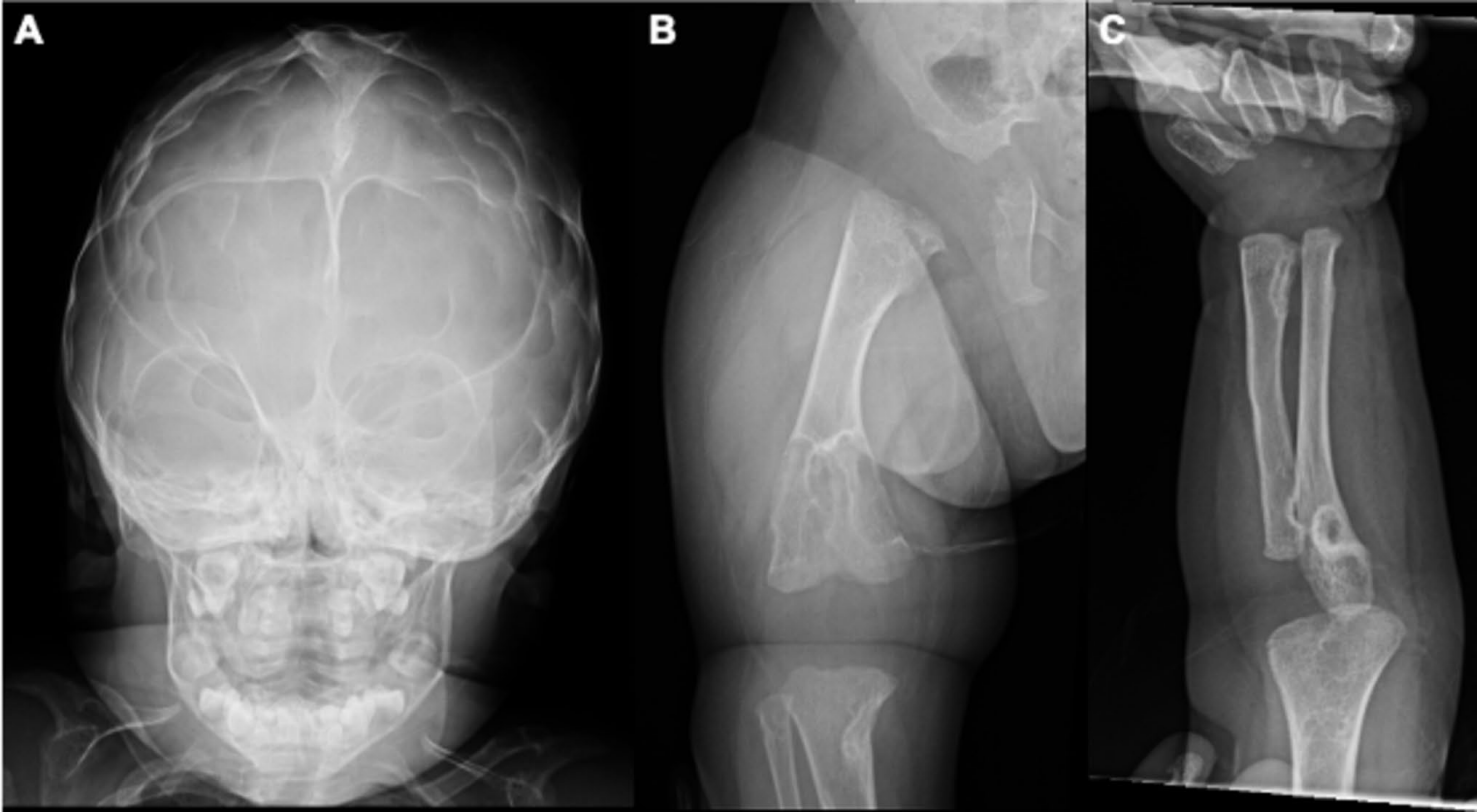
An 18-month-old with osteoglophonic dysplasia has radiographs demonstrating (**A**) “copper beaten skull” appearance from craniosynostosis, and nonossifying fibromas in the (**B**) distal femur, proximal tibia and fibula, and (**C**) the radius, ulna and humerus

Osteoglophonic dysplasia is a rare autosomal dominant syndrome of rhizomelic dwarfism associated with craniofacial features (prominent supraorbital ridge, depressed nasal bridge, choanal atresia or obstruction, and craniosynostosis), overlapping toes, pathologic fractures, dysplastic ossification centers, along with nonossifying cystic or lytic appearing skeletal lesions (Fig. 2). Hypophosphatemia has been reported in about 17% of patients with osteoglophonic dysplasia, with low 1,25(OH)_2_D levels, along with elevated FGF23 levels [18–20]. Osteoglophonic dysplasia is caused by pathogenic missense gain-of-function variants in FGFR1 involving the ligand binding and transmembrane domains of the receptor. Thus, FGFR1 activation is involved in the pathophysiology of increased FGF23 expression and resultant hypophosphatemia in this disorder, similar to that seen in TIO cases of FGF1 or FGFR1 fusion proteins [21].

## Autosomal Recessive Hypophosphatemic Rickets-FGF23 Mediated

### *DMP1* Variant ARHR

DMP1-related rickets (autosomal recessive hypophosphatemic rickets type 1, ARHR1) was independently described by two teams in 2006, in five unrelated kindreds [22, 23]. Roughly a dozen families with this rare FGF23-mediated form of rickets have been described to date [24–28]. Clinically, they present very similarly to XLH with rickets and associated skeletal bowing deformities in the lower limbs, growth impairment, and frequently with severe dental abscesses. Corriedale sheep represent a natural animal model of the disease [29]; studies in mouse models suggest abnormal phosphate sensing with a lower set point for phosphate in bone cells, leading to increased *FGF23* expression [30]. DMP1 deficiency leads to FGF23 excess by activating FGFR1 signaling in osteocytes [31]. Clinically, this has been treated similarly to XLH with active vitamin D (e.g., calcitriol) and phosphate salts. More recently, burosumab, a fully humanized monoclonal antibody against FGF23, has anecdotally led to clinical improvement in one patient [27].

### *ENPP1* Variant ARHR

**Fig. 3:**
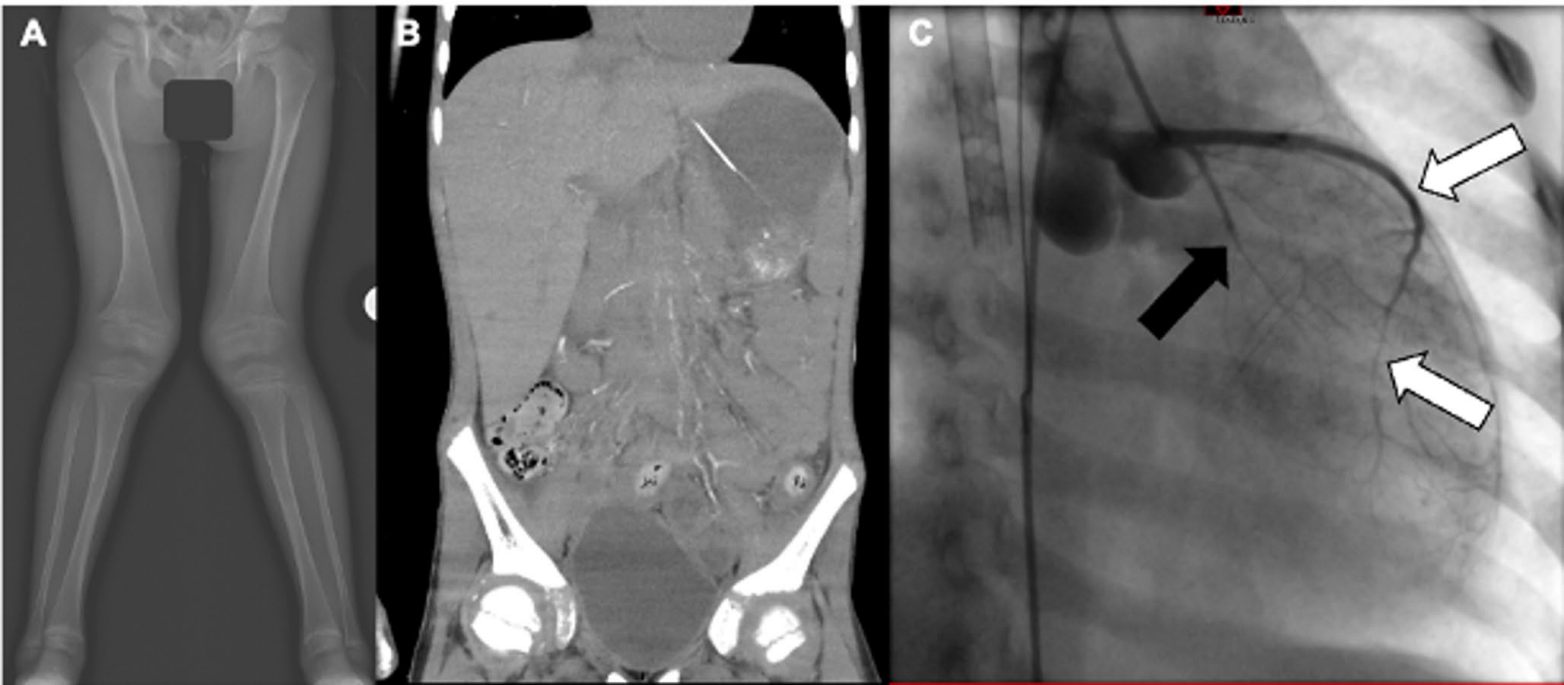
(**A**) Radiographs of a child with ENPP1 deficiency at age 4 years 11 months demonstrated genu valgus deformity and rachitic changes at the growth plate of the distal femur, proximal and distal tibia and fibula. (**B**) Abdominal CT of the same child at age 6 years 5 months demonstrating mesenteric artery calcifications. (**C**) Selective left coronary artery angiogram showing diffuse distal left circumflex (black arrow) and left anterior descending hypoplasia (white arrows) and irregularity with near-obliteration of flow

ENPP1 deficiency leads to Generalized Arterial Calcification of Infancy (GACI), a severe phenotype associated with calcification of the internal elastic lamina and thickening of the intima of arteries in early life (in utero or in infancy), and also to autosomal recessive hypophosphatemic rickets type 2 (ARHR2) (Fig. 3). These two phenotypes are not mutually exclusive; GACI has a high mortality rate of roughly 50% before the age of 6 months [32], but those who survive this critical period are then at risk of developing FGF23-mediated rickets/osteomalacia. The average onset of hypophosphatemia (serum phosphate below −2SD for age) is 1.6 years of age, with an estimated 20% developing rickets by 2 years of age [33], and 70% by 10 years of age [34]. Patients with GACI typically present with respiratory distress, heart failure and hypertension; some patients first present with rickets with no prior diagnosis of GACI, although in several of these patients cardiovascular problems (cardiac valve calcification, valvular regurgitation or stenosis, hypertension) were subsequently found [34]. Adult patients develop enthesis calcification and osteoarthritis, similar to XLH, with the majority of them experiencing residual pain despite the use of NSAIDs, and having impairment in physical function, most commonly of moderate severity [35]. FGF23-mediated osteomalacia has been described in patients with monoallelic variants in *ENPP1* [36, 37], although it is unknown if this is because specific heterozygous variants are severe enough to lead to skeletal disease in the absence of cardiovascular disease, or whether a second variant was missed by current genomic technologies. Similarly, enthesis calcification, including calcification of the spinal ligaments, has been described in monoallelic ENPP1 deficiency [37], although a similar phenotype also occurs in other disorders caused by FGF23 excess [38]. The cause of FGF23 excess in ENPP1 deficiency remains unknown, although the authors suspect disrupted purinergic signaling from ATP excess (as ENPP1 cleaves ATP into AMP and pyrophosphate), and are currently performing studies to confirm this hypothesis.

Bisphosphonates have been used in the treatment of GACI, though more recent evidence indicates there is not a significant survival benefit except possibly if started in utero or in the first week of after birth [32]. ARHR2 can be treated with supplemental phosphate and calcitriol; judicious treatment has been used safely without worsening of cardiovascular calcification [39], although aggressive treatment can lead to aggravation or recurrence of calcification [40]; regardless, this treatment is onerous and is associated with compliance issues due to the frequent doses of phosphate required. Burosumab should be avoided in this condition, as it has anecdotally been shown to lead to worsening of cardiovascular calcifications after 20 months of treatment [41]. Preclinical studies in mouse models of the disease have shown that enzyme replacement therapy with a recombinant form of ENPP1 leads to prevention of death and decrease of cardiovascular mineralization [42], improved cardiovascular function [43], prevention (before onset) or improvement (after onset) of arterial stenosis [44], improvement of bone mass, skeletal mineralization, and bone mechanical strength [45], and partial prevention of enthesis mineralization [35]. These promising preclinical results have led to current efforts to evaluate efficacy in clinical trials in humans.

### *FAM20C* Variant ARHR

FAM20C deficiency is associated with either a severe phenotype, as Raine syndrome, or a milder phenotype of autosomal recessive hypophosphatemic rickets type 3 (ARHR3). Raine syndrome is usually lethal in the neonatal period due to respiratory distress, and is associated with generalized osteosclerosis, brain calcifications, exophthalmos, severe midface retrusion, choanal atresia and a depressed nasal bridge. Survivors develop hypophosphatemia [46]. Patients with ARHR3 present first with FGF23-mediated rickets, along with generalized osteosclerosis, basal ganglia or periventricular calcifications, and early tooth decay as three important features distinguishing it from other causes of hypophosphatemic rickets [47–49]. Dental decay has been described as early as 18 months of age, often leading to edentulism before the age of 18 years [47]; tooth involvement includes periapical abscesses, dentin defects, and hypoplastic enamel. FAM20C phosphorylates FGF23, favoring its cleavage into inactive fragments by furin; thus, FAM20C deficiency leads to FGF23 excess by interfering with its inactivation [50].

### Opsismodysplasia

Opsismodysplasia is an autosomal recessive, sometimes lethal skeletal dysplasia associated with platyspondyly, metaphyseal cupping, delayed epiphyseal mineralization, and characteristic facial features (midface retrusion with depressed nasal bridge). Severe renal phosphate wasting was described in 5/9 patients in one of the original cohorts describing the genetic basis [51], and in 7/10 patients in a recent review [52]. This phosphate wasting is mediated by FGF23 [53]. Phosphate replacement has been shown to lead to clinical improvement, including gains in walking ability [54], while bisphosphonates have been shown to improve bone mineral density [55]. Opsismodysplasia is caused by variants in *INPPL1*, encoding SHIP2; although the exact cause of FGF23 excess is not known, SHIP2 has been shown to attenuate FGF signaling in vivo [56].

## Somatic Causes of FGF23-Mediated Hypophosphatemia

There are also other variations on FGF23-mediated hypophosphatemia, where focal lesions are responsible for FGF23 excess. Fibrous dysplasia/McCune-Albright syndrome (FD/MAS) is a mosaic skeletal disorder caused by gain-of-function variants in *GNAS*, encoding the stimulatory G protein alpha subunit. Approximately half of patients with FD/MAS have renal phosphate wasting (24/49) caused by FGF23 excess [57], with a strong relationship between total body FD burden and FGF23 levels. FD lesions have increased FGF23 expression, though levels are attenuated by decreased activity of GALNT3 and increased activity of furin, with concurrent increased FGF23 cleavage into inactive fragments [58]. Despite this increased FGF23 cleavage [58], frank hypophosphatemia is still possible. In a large cohort of patients, 20% (48/239) had frank hypophosphatemia (Z-score ≤ −2), 28% (66/239) had low-normophosphatemia (> −2 to ≤ −1), and 52% (125/239) had high-normophosphatemia (> −1 to ≤ 2); both frank hypophosphatemia and low-normophosphatemia were associated with increased skeletal morbidity [59].

The association between nevus sebaceus (a type of epidermal nevus) and hypophosphatemic rickets was made as early as the 1960 s [60]; by 2005, FGF23 excess was identified as the cause of hypophosphatemia [61, 62]. In 2014, the disorder was found to be caused by gain-of-function variants in *NRAS* (p.Gln61Arg) or *HRAS* (p.Gly13Arg), and the association was given the name of cutaneous-skeletal hypophosphatemia syndrome (CSHS) [63]. The cutaneous manifestations of this mosaic RASopathy include epidermal nevus in 61% of patients (31/51), phakomatosis pigmentokeratotica in 31% (16/51), and giant congenital melanocytic nevus in 8% (4/51) [64]. The source of excess FGF23 is the dysplastic bone carrying the somatic variant, not the cutaneous mosaic lesion [65]. Clinical improvement has been noted during treatment with burosumab [66] or with a MEK inhibitor, trametinib [67].

Tumor-induced osteomalacia (TIO) is caused by phosphaturic mesenchymal tumors (PMT). Approximately half of all PMTs carry gene fusions leading to FGFR1 overactivity, either through *FN1:FGFR1* fusion in 42% of cases (21/50) or *FN1:FGF1* fusion in 6% (3/50) [21]. In turn, around half (16/33) of PMTs that do not carry either aforementioned fusions instead harbor rearrangements involving the gene encoding Klotho [68]. The cause of FGF23 excess is thought to derive from direct FGFR1 overexpression (*FN1:FGFR1* fusion, as fibronectin drives the expression of FGFR1 in mesenchymal tissues), secreted FN1:FGF1 ligand activating FGFR1 via an autocrine loop, or overexpression of Klotho enabling an FGF23-FGFR1 autocrine positive feedback loop.

## Other Genetic Causes of non-FGF23-mediated hypophosphatemia

Patients with renal Fanconi syndrome have hypophosphatemia with normal levels of FGF23 [69]. Various inheritance patterns occur, including autosomal recessive (nephropathic cystinosis [70], NDUFAF6 deficiency [71]), autosomal dominant (EHADDH deficiency [72], GATM deficiency [73]), X-linked (Lowe syndrome, Dent disease [74]), and mitochondrial [75, 76].

A selective loss of the renal phosphate transporters (NaPi2a, encoded by *SLC34A1*, and NaPi2c, encoded by *SLC34A3*) also leads to renal phosphate wasting. While generally these have been considered to be recessive, there is evidence of frequent phenotypic manifestations in heterozygous patients as well. In patients with *SLC34A3* pathogenic variants, causative of hereditary hypophosphatemic rickets with hypercalciuria (HHRH), the frequency of isolated kidney phenotypes (nephrolithiasis, nephrocalcinosis, or renal cysts) was 24% (22/90), the presence of isolated bone phenotypes (rickets/osteomalacia, low BMD, or fractures) was also 24% (22/90), and the frequency of combined renal and bone phenotypes was 43% (39/90) in patients with biallelic disease, whereas for patients harboring a single heterozygous variant, 27% (38/141) had an isolated renal phenotype, 11% (16/141) a bone-only phenotype, and 11% (16/141) a combined phenotype [77]. Patients with biallelic *SLC34A3* variants present at a median age of 8.1 years, and patients with heterozygous variants at a median age of 5.0 years [78].

Patients with *SLC34A1* variants present at a younger age, diagnosed in infancy for those with biallelic variants (median age 4.8 months) and at a median age of 2.5 years for those with heterozygous variants. Patients with biallelic *SLC34A1* variants presented with polyuria (38%, 8/21), growth failure (32%, 7/22), vomiting (27%, 6/22), and constipation (23%, 5/22), while patients with heterozygous variants presented with flank or abdominal pain in 29% of individuals (7/24), constipation in 20% (5/25), hematuria in 16% (4/25), and growth failure, vomiting, and polyuria each in 12% (3/25) [78]. Hypercalcemia is found in 36% and 8% of patients with biallelic *SLC34A1* and *SLC34A3* variants, respectively, whereas hypophosphatemia and decreased TmP/GFR are found in 22% and 54% of patients with biallelic *SLC34A1* and *SLC34A3* variants, respectively (both p < 0.0.5) [78].

Finally, familial causes of hyperparathyroidism also account for inherited non-FGF23-mediated hypophosphatemic disorders, though typically having hypercalcemia instead of rickets [79].

In a study of 149 patients with hypophosphatemia, of whom 130 had FGF23-mediated hypophosphatemia (40 with TIO, 36 with XLH, 36 with FD/MAS, 12 with ENPP1 deficiency, 5 with CSHS, and 1 with NF1) and 19 had FGF23-independent hypophosphatemia (16 with cystinosis, 1 each with GATM-related familial Fanconi syndrome, Lowe syndrome, and HHRH), intact FGF23 was shown to be superior to C-terminal FGF23 in differentiating FGF23-mediated hypophosphatemia, with an intact FGF23 cut point of 27 pg/mL being 100% sensitive and specific in distinguishing FGF23-mediated from FGF23-independent hypophosphatemia [17].

## Non-genetic Causes of FGF23-mediated Hypophosphatemia

An autoimmune cause of hypophosphatemia was recently identified in five individuals with antibodies against PHEX, leading to mild elevations of intact FGF23 (mean: 55.6 pg/mL); only one of the patients had other concurrent autoimmune diseases [80].

Ectopic FGF23 production by cancer cells is another potential cause of hypophosphatemia. A review of such cases with renal phosphate wasting found that the most common associations included prostate cancer (26/52 reports, 50% of cases), lung cancer (11/52, 21%), and hematologic malignancies (4/52, 8%) [81]. Hypophosphatemic osteomalacia can be seen as a result of cadmium toxicity in silverware industry workers [82, 83]. Cadmium has been shown to increase FGF23 levels in mice by stimulating *GALNT3* expression and thus decreasing FGF23 cleavage [84, 85].

FGF23 excess with profound hypophosphatemia have also been found in patients with acute hepatitis, mainly from alcoholic hepatitis but also from erythrocytic protoporphyria. In these cases, excessive hepatic production of FGF23 was demonstrated in human and mouse liver biopsies [86, 87]. Similarly, hypophosphatemia may result from liver production of FGF23 as implicated in some cases of biliary atresia, including some that were cured after liver transplant [88, 89].

After a renal transplant, 85–90% of patients experience hypophosphatemia as a result of “tertiary hyperphosphatoninism”, a situation where FGF23 excess pre-transplantation persists and causes low phosphate and calcitriol levels in the early post-transplant period [90, 91]. Although the hypophosphatemia usually resolves within the first few weeks to months after the transplant, in some patients renal phosphate wasting may persist for several months [92].

As described above, in iron-deficient states, *FGF23* gene expression increases resulting in high C-terminal FGF23 (which includes fragments) but intact FGF23 remains normal. However, iron administered intravenously often causes acute rises in intact FGF23 and hypophosphatemia. The cause of iron infusion induced FGF23 excess remains unknown, but is thought to be dependent on effects of the carbohydrate moiety to possibly acutely impair cleavage of FGF23 (whether directly or indirectly). Intravenous formulations of iron for the treatment of iron-deficiency anemia are composed of an iron core surrounded by a carbohydrate shell to decrease the chance of infusion reactions. The risk of FGF23-mediated hypophosphatemia varies with the specific formulation of intravenous iron, but some risk for hypophosphatemia is present with all formulations. The risk is highest with ferric carboxymaltose, with the frequency of moderate hypophosphatemia (≤ 0.64 mmol/L) varying from 59.2–74.4%, severe hypophosphatemia (≤ 0.32 mmol/L) from 11.3–13.7%, and prolonged hypophosphatemia (≤ 0.64 mmol/L until the end of study) from 4.7–37% in patients without chronic kidney disease [93]. By comparison, ferric derisomaltose was associated with a frequency of moderate hypophosphatemia of 3.9–12.5%, with no reported cases of severe or prolonged hypophosphatemia [93]. Several reports have indicated that repeated administration of intravenous iron for iron deficient patients may cause hypophosphataemic osteomalacia, with consequences of bone pain, fractures and muscular weakness [94–97].

Of note, C-terminal fragments of FGF23 have been postulated to contribute to regulation of erythropoiesis, and administration of fragments blocks hepcidin induction to increase iron [98]. Additionaly, among patients with gain-of-function variants in *HIF2A*, which generates high erythropoietin, there are elevations of FGF23 fragments, but through normal cleavage, normal intact FGF23 and phosphate levels persist [99]. These scenarios indicate that FGF23 cleavage is a regulatable process and that disease results from dysregulation of this process.

## Conclusion

While XLH is more common, the several autosomal dominant, recessive, or other causes of hypophosphatemic rickets and osteomalacia deserve careful consideration in the evaluation of patients. Proper diagnosis is necessary to safely and effectively treat these patients.

## Key References

- Hartley IR, Gafni RI, Roszko KL, Brown SM, de Castro LF, Saikali A, et al. Determination of FGF23 Levels for the Diagnosis of FGF23-Mediated Hypophosphatemia. J Bone Miner Res. 2022;37(11):2174–85. https://doi.org/10.1002/jbmr.4702.
  - This article thoroughly assess both intact and C-terminal assays for FGF23 and experimentally provides threshold indicators to differentiate between FGF23 mediated and FGF23 independent causes of hypophosphatemia.
- Imel EA, Liu Z, Coffman M, Acton D, Mehta R, Econs MJ. Oral Iron Replacement Normalizes Fibroblast Growth Factor 23 in Iron-Deficient Patients With Autosomal Dominant Hypophosphatemic Rickets. J Bone Miner Res. 2020;35(2):231–8. https://doi.org/10.1002/jbmr.3878.
  - This pilot clinical trial demonstrated efficacy of oral iron repletion to normalize both FGF23 and phosphate in patients with *FGF23* variants causing ADHR.
- Ferreira CR, Kavanagh D, Oheim R, Zimmerman K, Sturznickel J, Li X, et al. Response of the ENPP1-Deficient Skeletal Phenotype to Oral Phosphate Supplementation and/or Enzyme Replacement Therapy: Comparative Studies in Humans and Mice. J Bone Miner Res. 2021;36(5):942–55. https://doi.org/10.1002/jbmr.4254.
  - This study demonstrated the skeletal response in humans and mice with ENPP1 deficiency to treatment with phosphate and calcitriol along with the risk of nephrocalcinosis, and in parallel studies that of the mouse model the improved skeletal and renal response to ENPP1 enzyme replacement therapy.

## Data Availability

No datasets were generated or analysed during the current study.
